# Influenza

**Published:** 1899-04-01

**Authors:** 


### The Hospital

A Journal of

The Medical Sciences and Hospital Administration.

Vol. XXVI.—No. 653. | Edited by Sir Henry Burdett, K.C.B., and Solomon C. Smith, M.D., M.R.C.P. | [April 1, 1899.

### INFLUENZA.

The prevalence of the so-called "influenza," the real nature and cause of which, notwithstanding all the light thrown upon epidemic diffusions by modern bacteriology, seem so far wholly to have escaped detection, is once again becoming a somewhat serious consideration in many localities, more especially in regard to the extent to which it has become associated with or followed by pneumonia, often of a severe and dangerous type. One of the latest outbreaks is that which was recorded last week from H.M.S. "St. Vincent," Commander T. H. Fisher, R.N., a training ship for boys stationed at Portsmouth, which had already suffered severely on a previous occasion. It seems from the official report that the disease reappeared among the boys last week, and that on Wednesday one of them was attacked, while drilling with others on Whale Island, with such severity that he was conveyed to Haslar Hospital instead of to his ship, and that he died on the following morning. The narrative is too scanty to afford any trustworthy information with regard to the actual cause of death, but it is at least ascribed to influenza, and has occasioned the removal of all the boys, and their division between H.M.S "Victory" and H.M.S. "Achilles," until the "St. Vincent" has been subjected to a complete process of fumigation and disinfection. On the whole, we think, the present epidemic does not appear to have been characterised by severity, as far as the original symptoms are concerned; insomuch that not a few sufferers have been betrayed into imprudence by their very mildness, and have had only too much occasion to deplore the consequences of an attempt to struggle against the inevitable. If the experience of the last three or four years has taught anything, it is that the first appearance of influenza should mean complete surrender; and that bed is the only safe place until a normal temperature has been restored, and has been maintained for a sufficient time to afford security against the probability of a relapse. The obituary lists of the daily papers for the last few weeks have contained a dismal total of mortality ascribed to "pneumonia," and it is not too much to say that nearly all of this has been a consequence of neglect of the early stages of influenza, or of endeavours to return to the duties of life before complete recovery had occurred. We published last week a summary of Dr. Gee's highly interesting Lumleian lecture upon the infective nature of "common cold," and there can scarcely be a doubt that much of what he there advanced would be at least equally applicable to influenza. The more serious malady, in fact, seems to fade into or to be developed from the less serious by almost imperceptible gradations; and the diagnosis between them is not to be made either with certainty or with facility. It seems to be ascertained that assemblages of human beings, however occasioned, are eminently favourable to the development and extension of both, and it is probably for this reason that we have heard, during the last few weeks, of so many large establishments—commercial, educational, and other, not even excepting the House of Commons—being seriously crippled by the ravages of the disease. It is probable that few assemblages, in this respect, have more to answer for than those which occur in churches and chapels, not only on account of the imperfect provision for ventilation to which Dr. Gee has called attention, but also on account of the gathering together of persons whose power of resistance has been diminished by recent illness or by other circumstances. Among these, as affecting the two great bodies of Roman Catholics and Ritualist Churchmen, it cannot be questioned that the season of Lent has to be considered, or that it produces, in many people, an impaired capacity to make head against the in[illegible]sion of disease. Such an effect, moreover, would be especially prone to occur in a year in which the season of Lent occurred early, and in coincidence with weather of unusual coldness and inclemency. Thackeray's curate, who had "taken a parched pea after matins," is not without parallels in our own day; and it would probably not be difficult to track him and his like through the progress of many invasions of the disease. For people possessed of common sense, the obvious lesson to be deduced from experience is tolerably plain. It is to avoid large assemblages and ill-ventilated places generally, to wear a sufficiency of warm and light clothing, to avoid chills, over-fatigue, and unwholesomeness of all kinds, and finally, if stricken, to throw up the sponge, to go to bed, and to stay there until the temperature has been normal for at least twenty-four hours. The chief danger which now besets the influenza patient arises from the not unnatural impatience of the sick man, and from his desire to resume his ordinary activities at an earlier period than either experience or skill could sanction.

